# Acoustic analysis of vowel formant frequencies in genetically-related and non-genetically related speakers with implications for forensic speaker comparison

**DOI:** 10.1371/journal.pone.0246645

**Published:** 2021-02-18

**Authors:** Julio Cesar Cavalcanti, Anders Eriksson, Plinio A. Barbosa

**Affiliations:** 1 Department of linguistics, Stockholm University, Stockholm, Sweden; 2 Institute of Language Studies, Campinas State University, Campinas, Brazil; Leiden University, NETHERLANDS

## Abstract

The purpose of this study was to explore the speaker-discriminatory potential of vowel formant mean frequencies in comparisons of identical twin pairs and non-genetically related speakers. The influences of lexical stress and the vowels’ acoustic distances on the discriminatory patterns of formant frequencies were also assessed. Acoustic extraction and analysis of the first four speech formants F1-F4 were carried out using spontaneous speech materials. The recordings comprise telephone conversations between identical twin pairs while being directly recorded through high-quality microphones. The subjects were 20 male adult speakers of Brazilian Portuguese (BP), aged between 19 and 35. As for comparisons, stressed and unstressed oral vowels of BP were segmented and transcribed manually in the Praat software. F1-F4 formant estimates were automatically extracted from the middle points of each labeled vowel. Formant values were represented in both Hertz and Bark. Comparisons within identical twin pairs using the Bark scale were performed to verify whether the measured differences would be potentially significant when following a psychoacoustic criterion. The results revealed consistent patterns regarding the comparison of low-frequency and high-frequency formants in twin pairs and non-genetically related speakers, with high-frequency formants displaying a greater speaker-discriminatory power compared to low-frequency formants. Among all formants, F4 seemed to display the highest discriminatory potential within identical twin pairs, followed by F3. As for non-genetically related speakers, both F3 and F4 displayed a similar high discriminatory potential. Regarding vowel quality, the central vowel /a/ was found to be the most speaker-discriminatory segment, followed by front vowels. Moreover, stressed vowels displayed a higher inter-speaker discrimination than unstressed vowels in both groups; however, the combination of stressed and unstressed vowels was found even more explanatory in terms of the observed differences. Although identical twins displayed a higher phonetic similarity, they were not found phonetically identical.

## Introduction

The present experimental study aimed at assessing the inter-speaker discriminatory potential of vowel formant frequencies in comparisons of genetically-related (i.e., identical twins) and non-genetically related speakers in Brazilian Portuguese (BP) from a forensic phonetic perspective. The speaker-discriminatory potential of F1-F4 formants was evaluated while observing how consistent these acoustic-phonetic parameters were when the limits of inter-speaker variation are considerably narrowed. In addition, the relation between vowel quality and inter-speaker variability was explored to identify which set of vowel segments are considered optimal for the forensic speaker comparison (FSC) task in BP. Furthermore, the acoustic dispersion and distances between BP cardinal vowels were inspected in order to observe how these factors may relate to vowel formant frequency variation.

In the past few decades, attempts have been made towards a proper explanation as to whether human voice and speech features are endowed with absolute specificity. From a theoretical point of view, most of the experiments addressing individuality in voice and speech have tried at some level to shed light on the relevant and challenging question “does each person in the population have a measurably unique voice?” [1]. Some studies have presented an answer to this question over the past few years (e.g., [2–8]), which corroborate this specificity at some level, with the magnitude of differences depending on the subjects under analysis.

Assessing aspects of individuality in speech and the limits of phonetic variation between very similar individuals require a considerably high experimental control level. As such, distances between individuals due to biological (*nature*) and environmental (*nurture*) factors have to be considerably shortened and controlled. In response to this challenge, identical twins have been the focus of scientific experiments in many different fields, such as medicine, genetics, psychology, anthropology, and linguistics, which has resulted in a scientific approach widely known as the *twin method*.

As described by [9], the *twin method* is founded on the biologic fact that monozygotic twins (MZ), the so-called identical twins, originate from the division of the same zygote. As a result, they are considered to be genetically identical. It follows that any phenotypic differences between MZ twins must be explained by environmental influences, expressed by any factors that are not *a priori* fixed genetically. Conversely, dizygotic twins (DZ), also known as fraternal twins or non-identical twins, like any other siblings, share only half of their genes.

In terms of structural or anatomical aspects, identical twins are assumed to have very similar vocal tracts in size and shape. According to [10], studies of genetically identical speakers make it clear that a person’s genetic makeup is a major factor in determining their overall size, shape, growth rate, and maturation. Moreover, there is evidence of genetic influences on structures of the brain. As reported by [11], genetically identical twins are almost entirely correlated in their gray matter distribution, including areas related to language cortices, for instance. This brings into question the rather complex relationship between *shape* and *function*.

As highlighted by [4], investigations on the speech patterns of twins from a forensic phonetic perspective allow researchers to understand the very limits of variation between speakers. The fact that most of these individuals presented similar linguistic and environmental influences reflects a considerable reduction of common sources of inter-speaker variation, allowing the assessment of phonetic parameters’ consistency.

### Variability in vowel formant frequencies

Formant frequencies are considered one of the most frequently assessed parameters in the forensic speaker comparison (FSC) practice. In general, the FSC task is commonly carried out considering F1, F2, and F3, due to the inaccessibility imposed by the telephone bandwidth to higher frequencies [12]. Notwithstanding, recent technological advances in telephone communication (e.g., WhatsApp and Telegram) have widened the possibilities of using higher frequencies, such as F4 and F5, in evidence materials [13]. In view of that, more experimental studies on vowel formant frequencies from a general forensic-phonetic perspective are needed.

In terms of speech production, vowel segments—as other speech sounds—are believed to convey information of three different dimensions: linguistic, social, and idiosyncratic dimensions, as mentioned by [14] in a widely cited study. These dimensions are directly related to the variation in speech and responsible for shaping ones’ “speech profile” at the acoustic, articulatory, and perceptual levels.

As pointed out by [14], linguistic information refers to what is being said or “the significance of the utterance” conveyed through the shared linguistic system. Together with the linguistic content, there is information related to the general background of a speaker, such as geographical origin, social class, social groups, level of education. These aspects are related to the social or socio-linguistic dimension. A third kind of information can also be identified, namely the idiosyncratic features of a person’s speech, expressed by learned speech patterns acquired throughout life and by anatomical and physiological aspects, dependent on the shape of the vocal tract, its dimensions, and proportions. In this sense, both the linguistic and the socio-linguistic information conveyed by vowels might depend mainly on the relative positions of the formants or, in the authors’ terms, on “the relative formant structure of the vowels”. In contrast, personal information seems to depend partly on the absolute values of the formant frequencies, given that the frequency ranges in which someone speaks cannot be modified by natural means as they are related to anatomic and physiological properties. In this sense, physiological and anatomical factors are acknowledged sources of inter-speaker variation concerning vowel formant frequencies.

Other sources of inter-speaker variations are commonly reported in the literature, including differences as a function of sex, dialect, age, speaking style, and speech rate. For instance, sizes of the F1 and F2 spaces tend to be larger for women than for men, and formant values are considerably higher for females when compared to males [15–17], which is also due to anatomical reasons, as for the descent of the larynx in males during puberty [18]. Moreover, differences in F1 and F2 formant frequencies have also been found during the lifespan, in which both formant frequencies and bandwidths tend to decrease during typical speech development. Additionally, decreases in voice fundamental frequency and F1 due to aging are reported to be more likely in females than males [17]. There is also evidence pointing to a higher inter-speaker variability of acoustic vowel spaces between slow and fast speakers, with slow speakers displaying a larger average variability [19], and differences related to speaking style, with a tendency to more centralized formant values in spontaneous speech when compared to word reading, for instance, [20]. Other linguistic factors have also been found to influence F1 and F2 values, and consequently, the dispersion of vowels in the vocalic space, such as the effect of lexical stress. In that regard, [21] observed that lexical stress has different effects on different vowels in Hebrew, with the vowels /a/ and /e/ being the most clearly affected, with a tendency of centralization in the unstressed condition. The same centralization tendency was observed by [22] for the Spanish vowels /a/ and /o/, and by [23] for the Brazilian Portuguese vowel /a/, implying a higher dispersion of the matching stressed vowels in the vocalic space in such languages. Furthermore, a combined effect between speech tempo and stress on the overall size of vowel spaces has been verified by [24] in American English, with a tendency for larger vowel spaces for the slow stressed condition and smaller for the fast unstressed condition.

In terms of vowel acoustic specification, the quality of a vocalic segment is primarily correlated to the frequency of the first and second speech formants [25], namely F1 and F2, produced by the proper manipulation of mouth opening and constriction location. Variations in these dimensions are related to the degree of articulatory precision required for producing a given vowel [26]. Furthermore, higher formants such as F3 (i.e., somewhat related to vowel configuration, as in the case of front rounded vowels) and F4 are commonly referred to as being considerably speaker-specific, conveying more speaker discriminant information [13]. These formants are also seen as related to voice quality aspects in spoken and singing voice [27].

According to [18], the position of the higher formants in the spectrum, as of F3, and F4, is largely determined by the vocal tract length. Moreover, in the experiment conducted by [26], the authors observed that while F2 tended to increase in frequency as the point of constriction moved forward from the glottis, there was only a small increase in F3 as the mouth opening increased in size and became less rounded during the referred movement. In general, the rate of the increase depended mostly on the size of the constriction.

Regarding the resonance production in the vocal tract, both one-dimensional and three-dimensional acoustic experiments confirm that the fourth formant frequency (F4) is generated in the proper larynx. The experiments also suggest that the laryngeal cavity (LC) is acoustically independent of other parts of the vocal tract [28, 29]. As reported by [29], experiments with acoustic models showed that the elimination of the LC also resulted in the suppression of F4 while retaining other formants. The same study also indicated that F4 is considerably sensitive to LC shape changes, as in the case of constrictions in the ventricular area, which increase F4 while other formants remain nearly stable. In the same direction, the experiment carried out by [13] with sustained monophthongs also had shown that when F4 increased across different speakers, only F5 seemed to follow in most situations, revealing a positive correlation between these formants.

In light of that, the present study represents an attempt to deepen the investigation on vowel formant frequency variability from a forensic-phonetic perspective while providing control for common inter-speaker variation sources, such as physiological and environmental factors.

### Vowel formant frequency analysis in twin pairs

Relatively few studies have investigated the speech or voice characteristics of genetically-related speakers using acoustic analysis, as in MZ twins. Most of the research has been carried out with English-speaking subjects, limiting the possibilities of cross-language comparisons. Other languages, however, have been addressed in recent years, such as German [8, 30–32], European Spanish [6, 7, 33, 34], Shanghainese and Mandarin [35]. It is also relevant to mention that small-sized studies are persistent in research involving identical twin speakers, which does not invalidate the observations but suggests some caution regarding generalizations.

In a pioneer study, [1] looked at vowel formant frequencies in a group composed of identical twins. In their analysis carried out with three pairs of identical twins, aged between 20 and 23, who had grown up together, it was possible to find discriminable differences in controlled speech material. The experiment was performed with readings of a word list containing /l/ and /r/ before vowels in words such as “lip”, “let”, “lot”, “lug”, “rip”, “rap”, and “rock” of Southern British English. In the experiment, variations for F1, F2, F3, and F4 were observed for specific twin pairs, which according to the authors, suggest that twins are not necessarily phonetically identical and can appropriate themselves of the same articulatory freedom as other speakers to opt for alternative phonetic realizations.

The first research in Brazilian Portuguese, which approached identical twins within the forensic phonetic perspective, was conducted by [36]. In this study, the vowel formant frequencies (F1-F4) of only one identical twin pair were assessed through reading. The main results suggested that the mean formant frequency of the twins was considerably similar, especially for F2 and F4. Conversely, the identical twin pair was found to behave differently for F1 and F3, as evidenced by the statistical analysis. The results also indicated a significant interaction in both F1 and F3 regarding the variables “speaker” and “vowel quality”, with only the nasal vowel [$\tilde{ɐ}$] displaying significant differences. According to [36], it is likely that the speakers revealed different strategies for nasal quality production, involving, for instance, different degrees of velum opening.

In an acoustic and perceptual experiment with Australian-English vowels involving three pairs of similar-sounding MZ twins during a conversational speech task, [2] observed significant acoustic differences for the productions of /æ/ and /ᴧ/ in terms of F1. No significant differences were found regarding F2 and F3 for any of the eleven vowel segments analyzed. Concerning F4, significant differences were found for all vowels except for the back vowels /u/ and /Ʊ/. As argued by the author, the results cannot disprove the assertion that each person in the population has a measurably unique voice.

In a follow-up study carried out by [3], with the goal of analyzing the degree of speaker-specificity of Australian-English monophthongs in the speech patterns of four male twin pairs (three MZ and one DZ), it was observed that the highest discriminant parameters for this group were F2 and F3 of the close-front vowel /ɪ/, followed by F3 of the front vowel /æ/, F3 and F2 of the close front vowel /i/ and F3 of the front vowel /ε/. The results of this investigation have presented some clear evidence that front vowels in Australian-English were more speaker-specific than other vowels. Overall, the six most speaker-specific parameters in this study were from front vowels, and four of the five most speaker-specific parameters were from close-front vowels.

In the same direction, in [4], a very comprehensive acoustic study with static formant analysis was carried out with similar-sounding twin pairs, in which a forensically realistic material consisting of spontaneous conversational speech, composed by direct (four twin pairs) and telephone recordings (five twin pairs), as well as non-contemporaneous data were assessed. As in the previous one, an important aspect of this study regards the methodological approach applied, carried out with the analysis of same-segment vowel tokens, rather than strictly controlling to phonetic context. The formant analysis (F1-F4) of lexically stressed Australian English vowels also included a variability of phonetic contexts. In the referred experiment, differences in vowel realization were found where some speakers had consistently more fronted vowels than their twins, which has also been confirmed through acoustic analysis. A re-analysis of the data using a likelihood ratio (LR) approach confirmed that twins’ speech was much closer in F-pattern than pairs of unrelated speakers in the corpus. The results also revealed that the inter-speaker variation was greater than the intra-speaker variation concerning the parameters assessed.

Furthermore, an acoustic study was conducted by [37] with a pair of Southern Irish male MZ twins (T1 and T2) aged 21 years old and their age and sex-matched sibling, who participated in the experiment two years later. It was observed by the examination of F2 vowel onsets and targets that the MZ twin pair displayed F2 values and coarticulation patterns that were more similar than those of their age- and sex-matched sibling. According to the authors, the higher correspondence between MZ twins might be explained by greater physical similarities between the vocal tracts of T1 and T2 when compared to their sibling.

Later on, [30] explored the articulatory and acoustic inter-speaker variability in the production of German vowels in stressed and unstressed conditions. The vowels /i/, /u/, and /a/ were produced by two female and one male MZ twin pairs and two female DZ twin pairs, aged between 20 and 34. The formants F1-F4 of each vowel were measured and then compared within the pairs. The results demonstrated that the inter-speaker variability was equally distributed for two out of the three MZ pairs, regarding low (F1-F2) and high formant frequencies (F3-F4). In contrast, within the DZ twins, F1 and F2 accounted for approximately 35% of the differences. According to the author, since the size and form of the vocal tract have a strong influence on the higher formants of a speaker, MZ twins are expected to show less inter-speaker variability in F3 and F4 than for F1 and F2, depicting higher formants as being more dependent on physiology and less influenced by alternative articulatory strategies. Notwithstanding, a considerable number of higher formant differences were still found between MZ twin pairs.

Concerning the analysis of the lexical stress in [30], DZ twin pairs revealed more inter-speaker variability in unstressed than in stressed syllables. Conversely, two out of three MZ twin pairs showed no differences in the unstressed condition, only in the stressed context. According to the researcher, physiology seems to have a stronger influence on the production of a vowel when it is produced in an unstressed syllable (i.e., less acoustically salient).

From a dynamic perspective, there is also evidence of vowel formant transitions as being remarkably speaker discriminatory, with identical twins displaying consistent variations in the production of vocalic sequences, such as diphthongs [33–35].

Within a forensic-phonetic scope, [34] investigated the dynamic acoustic properties of 19 vocalic sequences of Standard Peninsular Spanish, assessing their potential for forensic speaker comparison while using curve-fitting estimated coefficients. The study was carried out with male MZ and DZ twins, brothers, and unrelated speakers, aged between 18 and 52. The experiment was designed in a way to elicit specific vocalic sequences during a collaborative task, namely, finding out the missing information in a fax copy. The main outcomes of this study were that the fusion of 19 vowel sequences outperformed the analysis carried out with individual transitions, and the geometric-mean combination method outperformed the logistic regression analysis. In the experiment, MZ twins were found to deteriorate the system’s performance for all vocalic sequences. Moreover, the fact that higher or lower similarities were found depending on the specific twin pair would indicate that the parameters assessed are not completely and uniquely genetically influenced, as claimed by the authors.

Despite the acknowledged high speaker-discriminatory potential of dynamic parameters, such as the analysis of formant transitions, some practical implications must be considered. From a forensic-phonetic perspective, it is relevant to mention that vocalic transitions may require more data to account for the variation observed among individuals. In contrast, monophthongs may be regarded as substantially more recurrent, as evidenced by the higher frequency of monopthongs in relation to diphthongs during the analysis of the present study. Also, for this reason, most of the studies carried out with dynamic parameters are undertaken with controlled speech material, as to prompt the very same vowel sequences across individuals, as in [35]; or require an ad hoc design to induce the intended sequences in spontaneous speech [34]. This factor may solely reduce the practical application of dynamic features in some forensic speaker comparison contexts.

It may also be relevant to mention that static and dynamic features can both help explain how different speakers are from each other, providing complementary and useful data. In [8], for instance, while MZ twins were found similar and DZ twins different for dynamic formant patterns (F2 and F3 transitions in sibilant-schwa sequences), when static parameters were included in the analysis, namely the spectral center of gravity, mean spectral peak, and mean formant measures, both MZ and DZ twins were found substantially different. According to the authors, physiological factors might present more influence on dynamic parameters, which was furthermore corroborated in [31], where higher similarities for looping trajectories in tongue movements were observed for MZ twins when compared to DZ twins and unrelated speakers for VCV sequences (vowel-consonant-vowel), as evidenced through electromagnetic articulography (EMA).

With regard to phonetic studies in Brazilian Portuguese, there is a lack of evidence concerning how robust and consistent vowel formant frequencies are when considerably similar speakers are compared. Additionally, very little is known about the limits of phonetic variation for related individuals, as genetically-related subjects. The present study aims to advance this understanding.

## Research questions and hypotheses

The present study aimed at answering the following research questions concerning Brazilian Portuguese speakers:

1. From a phonetic-acoustic perspective, is it possible to differentiate identical twin pairs through the analysis of vowel formant mean frequencies?
2. Are identical twins phonetically more similar than non-genetically related speakers?
3. Which set of parameters and vowel segments are considered more inter-speaker discriminatory in comparisons between genetically and non-genetically related individuals?
4. What are the effects of lexical stress on the discriminatory power of vowel formants?

Based on the exposed, a few hypotheses have been suggested: i. Identical twin pairs are expected to be substantially more similar than non-genetically related speakers (henceforth, NGRS). ii. It is expected that higher formant frequencies will be relatively more speaker discriminatory than lower formants. iii. It is predicted that the cardinal vowels displaying higher distances from their neighbors in terms of F1 and F2 might be the ones displaying the highest levels of phonetic variation. iv. Regarding the stress component, it is expected that stressed vowels may display a higher speaker-discriminatory power in identical twins, whereas unstressed vowels may display a higher inter-speaker variability for NGRS, based on previous literature findings [8].

## Method

The present study, which derives from the first author’s doctorate thesis, was registered under the protocol 95127418.7.0000.8142, evaluated and approved by the ethical committee at Campinas State University (UNICAMP). All participants voluntarily agreed to be part of the research verbally and by signing a participant consent form. All personal information regarding the participants is kept private.

### Participants

The participants are 20 subjects, ten male identical twin pairs consisting of Brazilian Portuguese (BP) speakers of the same dialect. The age range was from 19 to 35 years old, with a mean of 26.4 years. All identical twin pairs were codified with letters and numbers, such as A1, A2, B1, B2, C1, C2, D1, D2, and so on. The same letters indicate that the speakers are identical twins and therefore related.

It is worth noting that twin pairs were treated as their control group for comparisons among unrelated speakers, as done through cross-pairs comparisons (e.g., A1—B1; A1—B2; A2—B1, A2—B2, and so on). Overall, 190 comparisons among speakers were carried out for each tested parameter.

In the present study, the term “identical twins” will be employed over “monozygotic twins” for the sake of a practical aspect: the latter term implies the assessment of the twins’ genetic material. Since no laboratory genetic analysis was carried out, the first term will be therefore adopted. However, the use of the expression “identical” by no means implies that speakers are identical to each other; it solely refers to the pairs’ relatively high physical similarities.

The participants resided in five different cities within Alagoas State, which is the second smallest state in Brazil, with an area of 27.8 km². No substantial dialect variation between speakers was observed by the first author, who is a native speaker of the same dialect.

The subjects were selected through a recruitment method known as chain sampling, in which subjects are contacted among their acquaintances or by indication of other participants.

The inclusion criteria were: i. Identical twins; ii. male speakers; iii. same dialect; iv. aged between 18-45 years; v. with at least elementary school completed. The exclusion criteria were: i. Self-reported hearing loss or speech disorder, ii. identical twins raised apart; iii. identical twins that lived apart from each other for more than five years.

### Recording procedure

The recordings were carried out in silent rooms located in the cities where the twins resided. The speech material consisted of two different tasks: (A) a telephone conversation between the twins and (B) an interview between individual participants and the first author. The analysis presented in this paper, however, refers solely to the first task.

During task (A), the twin pairs were placed in different rooms, so they could not directly see or hear each other. The speakers were encouraged to start an informal conversation through mobile phones while being simultaneously recorded using a high-quality microphone.

The audio signals were then processed and registered in two separate channels. The referred recording approach aimed at eliciting a telephone speaking style and represents an attempt to approximate the experimental conditions to more realistic forensic circumstances, similarly to the experimental design proposed by [33] in a forensic phonetic study with twins.

All recordings were performed in high resolution, with a sample rate of 44.1 kHz and 16-bit quantization, using an external audio card (Focusrite Scarlett 2i2) and two headset condenser microphones (DPA 4066-B). The unedited recordings had an average duration of about 10 minutes. No audio degradation, such as telephone filter or background noises, were added to the original audio files, as the implications of telephone transmission will not be addressed.

The conversation topics were, in all cases, decided by the twin pairs before the recording sessions. Some general topics such as their childhood, events experienced by both, or themes related to their everyday life were suggested by the researcher, although the pairs were free to select any topics they preferred and could change it at any time.

It can be assumed that the familiarity effect between twin pairs was relatively well controlled in this experiment. The participants in the study were questioned about their social dynamic. They all reported having grown up together, sharing common social groups, being always in contact with each other, and displaying a high level of identification.

### Speech material

The focus of the present research lies at the seven vowels of BP /i, e, ε, a, ɔ, o, u/ extracted from speech samples corresponding to each of the 20 speakers recorded. Given the high occurrence of phonological processes in spontaneous speech, e.g., vowel harmony, phonetic vowels rather than phonemic segments are considered as the relevant empirical material.

The analyzed speech samples were about 2:30 minutes long and were extracted from different parts of the dialogues with the intention of increasing the data representativeness by capturing different speech patterns and trends throughout the recordings. Excerpts from the dialogues were selected from the initial, middle, and final parts, in portions where the dialogue contained fewer interruptions or conversation overlaps.

To the knowledge of the present authors, this is the first study in BP within the forensic phonetic domain with a group consisting of only adult identical twin speakers. One of the advantages of the present methodological approach is that it was carried out with a very representative corpus, with recordings of spontaneous conversational speech.

### Data extraction and analysis

All vowels were segmented and transcribed manually in Praat software [38] following auditory and acoustic criteria, namely the energy appearance/disappearance in the broad-band spectrogram. Nasalized vowels, diphthongs, and triphthongs were excluded from the corpus, resulting in a data-set composed only of oral monophthongs. The vocalic segments were then classified as stressed or unstressed as well as modal or creak by the first author, who is a certified speech-language pathologist. The analysis of creak events remains a topic for future investigation.

Since the recordings are of very high quality, the vowel labeling process was possible in most cases. The fact that all speakers were recorded simultaneously in different channels (i.e., eliminating speech overlaps that could disturb the labeling process) was also of great help. It is also noteworthy that the first author was present at the moment of the recordings, being able to base his judgment on the analysis of the speech context while performing the transcriptions. In some rare cases, vowel segments were excluded when their identity was too difficult to define.

The F1-F4 values were automatically extracted from the middle points in the labeled vowels through a Linear Predictive Coding (LPC) technique. The parameters extraction was done using a Praat script developed by the third author. The script generates a.txt file containing speaker identity, vowel names, vowel durations in seconds, and mean formant frequencies in Hertz. Given that extractions were carried out automatically and that only male voices were used, the hypothetical influence of extraction errors can be regarded as minimal.

For the comparison of identical twin speakers in the Bark scale (cf. [39]), formant frequencies in Hertz were transformed to Bark according to the following formula [40]:

$$\begin{array}{c} z=[26.81/(1+1960/f)]-0.53 \end{array}$$ (1)

Comparisons of formants in the Bark scale between identical twins were carried out in order to assess whether the observed differences could be perceptually or linguistically relevant.

Initially proposed by [39], Bark is a critical-band rate based on psychoacoustic principles, which are essential for understanding some characteristics of the human hearing system [41]. It is worth mentioning that not necessarily all differences observed in one scale are also significant in the other, once they are based on different acoustic principles. Also, it must be recognized that not all variations in speech production’s physical dimensions are relevant from a linguistic and perceptual viewpoint.

Furthermore, as to observe to what extent the acoustic distances of cardinal vowels in the vocalic space could be related to formant variation, the Euclidean distances between vowels were also assessed.

The Euclidean distances were measured by computing each segment’s F1 and F2 coordinates in the two-dimensional vocalic space. Subsequently, the mean acoustic distances between neighboring vowels and between the extreme front vowels (/i/—/ε/) and extreme back vowels (/u/—/ɔ/) were also quantified. The following formula was applied, in which (x, y) stands for the coordinates of vowel 1 in the Euclidean plane, whereas (a, b) stands for the coordinates of vowel 2 in the Euclidean plane.

$$\begin{array}{c} \text{dist}\left(\left(x,y\right),\left(a,b\right)\right)=\sqrt{(x-a{)}^{2}+(y-b{)}^{2}} \end{array}$$ (2)

### Statistical analysis

Since the present study’s data was found not fit into a normal distribution, as verified through the Shapiro-Wilk normality test, the statistical analysis was carried out with non-parametric statistical methods. The data were submitted to a Kruskal-Wallis rank sum test, followed by posthoc Dunn’s Multiple Comparison Test with Bonferroni correction (alfa/190), a two-tailed test.

Given that all speakers were compared for each vowel segment individually, the statistical significance threshold was calculated by considering the total number of comparisons carried out among individuals for each vowel segment, namely 190 comparisons in total. The p-value adjustment used, namely the Bonferroni correction, was performed automatically by the R software. As such, all p-values reported in the posthoc analysis have implicitly considered such correction.

As to assess whether stressed and unstressed vowels, as well as whether front and back vowels display different variances, the Fligner-Killeen test of homogeneity of variances was performed, which tests the null hypothesis that the variances in each of the groups are the same.

The discriminatory power of formants and vowels were represented by the number of statistically significant differences observed for each formant and vowel quality in relation to the number of comparisons carried out, that is, ten comparisons intra-twin pairs and 180 comparisons among NGRS, yielding a total of 190 comparisons across speakers. As such, the components displaying the highest number and proportions of inter-speaker differences would be regarded as more speaker-discriminatory.

Even though the statistical analysis was performed on the basis of the comparisons among all individuals, the reporting of the results took into account the separation between genetically-related individuals (i.e., identical twin pairs) and NGRS (i.e., cross-pair comparisons). This methodological approach prevented from treating one of these groups unequally in terms of the statistical method applied, avoiding possible methodological bias. As such, all differences reported here are based on and reflect the same statistical methods.

Finally, the effect sizes were computed for each vowel segment and each formant frequency as a function of inter-speaker differences. For the sake of verification, the effect sizes regarding (global) inter-speaker differences were also computed regarding each formant frequency. Moreover, intra-speaker differences were also assessed employing the Kruskal-Wallis rank sum test. For the assessment of intra-speaker differences, a four-point analysis was performed. This approach consisted of simply dividing all speech chunks into four more extensive speech excerpts, expected to mirror internal vowel production variations. Four mean values were derived from the different selection points, namely selection I, II, III, and IV. To ensure that all selections were representative of all dialogue parts, namely beginning, middle, and ending parts, and to reduce the effects of non-intended variations speech part-related, random chunks were picked to compose each selection.

For the estimation of the Kruskal-Wallis Effect Size, the following formula was applied, where H is the value obtained in the Kruskal-Wallis test; k is the number of groups; n is the total number of observations:

$$\begin{array}{c} \eta^{2}=\left(H-k+1\right)/\left(n-k\right) \end{array}$$ (3)

Furthermore, the magnitude of the differences observed were attributed automatically by the package ‘rstatix’, version 0.6.0, in the R software, in view of the values commonly reported in the literature for the eta-squared (η^2^): 0.01 ≤ 0.06 (small effect), 0.06 ≤ 0.14 (moderate effect), and ≥0.14 (large effect). The effect size index assumes values ranging from 0 to 1, which when multiplied by 100% indicates the percentage of variance in the dependent variable explained by the independent variable (cf. [42, 43]), in the present study: “speaker”.

## Results

A total of 9,446 vowels were analyzed in the present study, of which 5,487 (58%) were classified as stressed and 3,959 (42%) as unstressed. The most frequent vowels in the corpus were the central vowel /a/ with 3497 occurrences, /i/ with 1677, /u/ with 1116, /ε/ with 1015 and /e/ with 988 occurrences. The less frequent vowels were the back vowels /ɔ/ with 531 occurrences and /o/ with 622 occurrences. The number of vowel tokens by speaker varied from 402 to 588 tokens, a mean of 472 and a standard deviation of 61.8.

It may be noted that the three most frequent vowels were the most extreme and contrasting ones in the BP vocalic system, as revealed by the following occurrence order: /a/ > /i/ > /u/.

Considering that the vowels /ε/ and /ɔ/ are generally stressed in BP and produced as unstressed only in a few contexts for the analyzed dialect, a relatively small number of unstressed tokens for the corresponding segments was already predicted. Even so, all vowels were included in the analysis.

The reporting of vowel formant differences among speakers will consider the subdivision between vowel quality-related measures in BP—F1 and F2, and high formant frequencies —F3 and F4. Moreover, the articulatory phonetic distinction between front and back vowels will be acknowledged when reporting the differences. The main results are described in the following sections.

### Inter-speaker discriminatory potential of F1-F4 frequencies

Tables 1–4 present the statistically significant differences of mean formant frequencies observed across speakers. Differences are reported per individual vowel and per individual parameter (i.e., regardless of vowel identity). The overall percentage of differences observed for individual formants are presented in the column “%diff”, which considers the number of statistically significant differences for all seven vowels presented in “total”, in relation to the total number of comparisons carried out, in this case, 70 (10 x 7) for identical twin pairs and 1260 ((190-10)x 7) for NGRS. By calculating the totality of the differences observed for each formant, it is intended to virtually reduce the vowel quality effect on the parameter’s contrastive power. The same approach was applied to the analysis of vowels’ discriminatory potential.

**Table 1:** Significant (p<0.05/2) and marginally significant differences (p<0.10/2) within identical twin pairs for comparisons considering both stressed and unstressed vowels. Two-tailed test with Bonferroni correction.

	Front vowels				Back vowels				Differences
	i	e	ε	a	ɔ	o	u	Total	%Diff
**F1 (Hz)**	B1-B2 G1-G2		C1-C2	B1-B2 C1-C2 G1-G2	*G1-G2* *(p = 0.026)*	C1-C2		8	11%
**F2 (Hz)**	B1-B2 D1-D2 E1-E2 G1-G2	*D1-D2* *(p = 0.04)* *E1-E2*	B1-B2 E1-E2	B1-B2 E1-E2			*C1-C2* *(p = 0.04)*	11	15%
**F3 (Hz)**	D1-D2 E1-E2 G1-G2	C1-C2 G1-G2 (p = 0.03)	H1-H2	C1-C2 G1-G2 H1-H2	G1-G2 H1-H2		G1-G2 H1-H2	13	18%
**F4 (Hz)**	D1-D2 E1-E2 G1-G2 J1-J2	E1-E2 G1-G2 (p = 0.03)	C1-C2 E1-E2 G1-G2 H1-H2	C1-C2 D1-D2 E1-E2 F1-F2 G1-G2 H1-H2	E1-E2	E1-E2	E1-E2	19	27%
Total	13	6	8	14	4	2	4		
	Mean = 9				Mean = 3				Mean = 18%

**Table 2:** Number of significant differences (p<0.05/2) among non-genetically related speakers for comparisons considering both stressed and unstressed vowels. Two-tailed test with Bonferroni correction.

	Front vowels				Back vowels			Differences	
	i	e	ε	a	ɔ	o	u	Total	%Diff
**F1 (Hz)**	91	81	73	81	49	54	63	429	34%
**F2 (Hz)**	55	34	40	65	10	1	14	219	17%
**F3 (Hz)**	48	48	70	125	76	62	80	509	40%
**F4 (Hz)**	101	64	59	117	51	47	58	497	39%
Total	295	227	242	388	186	164	215		
	Mean = 254				Mean = 188				Mean = 33%

**Table 3:** Significant (p<0.05/2) and marginally significant differences (p<0.10/2) within identical twins for stressed and unstressed vowels. Two-tailed test with Bonferroni correction.

	**Stressed**								Differences
	**i**	**e**	**ε**	**a**	**ɔ**	**o**	**u**	**total**	**%diff**
**F1 (Hz)**	B1-B2		C1-C2	B1-B2 C1-C2 G1-G2		C1-C2		6	8%
**F2 (Hz)**	B1-B2 E1-E2	E1-E2	B1-B2 E1-E2	B1-B2 E1-E2			*J1-J2* *(p = 0.03)*	8	11%
Total	3	1	3	5	0	1	1		Mean = 10%
	**Unstressed**								Differences
	**i**	**e**	**ε**	**a**	**ɔ**	**o**	**u**	**total**	**%diff**
**F1 (Hz)**				C1-C2 G1-G2				2	3%
**F2 (Hz)**	D1-D2		E1-E2	*B1-B2* *(p = 0.026)* *E1-E2*				4	5%
Total	1	0	1	4	0	0	0		Mean = 4%

**Table 4:** Number of significant differences among non-genetically related speakers for stressed and unstressed vowels (p<0.05/2). Two-tailed test with Bonferroni correction.

	**Stressed**							Differences	
	**i**	**e**	**ε**	**a**	**ɔ**	**o**	**u**	**total**	**%diff**
**F1 (Hz)**	72	65	48	71	28	35	30	349	27%
**F2 (Hz)**	44	29	29	51	4	-	4	161	13%
Total	116	94	77	122	32	35	34		20%
	**Unstressed**							Differences	
	**i**	**e**	**ε**	**a**	**ɔ**	**o**	**u**	**total**	**%diff**
**F1 (Hz)**	42	19	23	53	7	12	25	181	14%
**F2 (Hz)**	17	2	5	32	-	-	-	56	4.5%
Total	59	21	28	85	7	12	25		9%

In the present study, the statistical analysis revealed consistent patterns regarding the comparison between low-frequency and high-frequency formants in identical twin pairs and NGRS, with high formant mean frequencies displaying a relatively higher discriminatory potential for both groups.

By consulting Table 1, it can be seen that the overall discriminatory power of the horizontal and vertical articulatory dimensions, assessed through F1 and F2, yielded a total of 11.5% and 15.5% of differences in twin pairs, respectively. Furthermore, as can be noted, identical twins appeared relatively more similar for F1 than for F2. When vowel specificity was considered, from a qualitative viewpoint, it has been found that five identical twin pairs contrasted significantly through the analysis of F1 and F2 for specific vowels (i.e., B1-B2; C1-C2; D1-D2; E1-E2; G1-G2).

As for the comparisons of NGRS, presented in Table 2, the inter-speaker comparisons through F1 and F2 yielded a percentage difference of 34% and 17.5%, respectively. As noted, NGRS were found more similar for F2 than for F1 in the present analysis. In general, the discriminatory potential of F1 appeared to be considerably higher for NGRS than for twin pairs, with a percentage difference of 22.5%. In contrast, the outcomes suggested a relatively similar discriminatory power of F2 when performing an inter-group comparison, with a percentage of 2% fewer differences for identical twin pairs compared to NGRS.

Regarding the comparisons of high formant frequencies as a function of vowel quality in twin pairs, both F3 and F4 parameters seemed to display a relatively higher discriminatory power. These parameters were able to differentiate seven twin pairs regarding some particular vowels (C1-C2; D1-D2; E1-E2; F1-F2; G1-G2; H1-H2; J1-J2), as presented in Table 1. Moreover, when the discriminatory power of formants was assessed independently, it was observed that identical twins appeared to be more distinct for F4 when compared to F3, with a percentage of 27% differences observed for the former and 18.5% for the latter. However, most of the difference between F3 vs. F4 seemed to be explained on account of the high F4 contrasting power regarding one pair: E1-E2.

As for the comparisons of F3 and F4 mean values among NGRS, a substantially similar contrastive power was suggested, of 40.5% and 39.5%, respectively. In an inter-group comparison, it was observed that NGRS also seemed to be considerably more distinct for F3 (22%) and for F4 (12.5%) in comparison to identical twin pairs.

Effect size estimates per individual vowel segment and formant frequency are displayed in Table 5, which serves as a statistical parameter allowing the assessment of the direction and strength regarding the relationship between variables [44]. By inspecting this Table, it can be verified that, for the comparisons carried out with the combination of stressed and unstressed vowel, only F4 displayed a large effect size for all vowels, followed by F3 and F1, which varied from mostly large to moderate effect sizes. In contrast, F2 displayed the smallest effect sizes, varying from mostly moderate to large effect sizes in front vowels to moderate and small effect sizes in back vowels. When comparing the global effect sizes regarding each formant frequency, it can be observed that only F3 and F4 displayed large effect sizes for inter-speaker comparisons, while both F1 and F2 displayed small global effect sizes. Moreover, given that no statistically significant differences were observed for intra-speaker comparisons (i.e., intra-selections), no effect sizes were computed.

**Table 5:** Effect size estimates for each vowel segment and each formant frequency.

Stressed and unstressed vowels								
	i	e	ε	a	ɔ	o	u	Inter-speakers
**F1**	0.237 large	0.312 large	0.253 large	0.106 mod	0.294 large	0.292 large	0.167 large	0.0363 small
**F2**	0.128 mod	0.133 mod	0.154 large	0.0965 mod	0.105 mod	0.0499 small	0.0560 small	0.0261 small
**F3**	0.0972 mod	0.174 large	0.284 large	0.346 large	0.566 large	0.365 large	0.294 large	0.191 large
**F4**	0.326 large	0.294 large	0.265 large	0.211 large	0.311 large	0.275 large	0.172 large	0.202 large
**Stressed vowels**								
**F1**	0.263 large	0.295 large	0.224 large	0.188 large	0.263 large	0.262 large	0.207 large	0.0312 small
**F2**	0.158 large	0.130 mod	0.174 large	0.159 large	0.0808 mod	0.0615 mod	0.0867 mod	0.0267 small
**Unstressed vowels**								
**F1**	0.209 large	0.397 large	0.330 large	0.102 mod	0.444 large	0.414 large	0.137 mod	0.0496 small
**F2**	0.108 mod	0.173 large	0.121 mod	0.0606 mod	0.203 large	0.0594 small	0.0385 mod	0.0273 small

Overall, the analysis of F1-F4 mean formant frequencies was able to contrast eight pairs of identical twins out of ten. Notwithstanding, two identical twin pairs were still considerably similar regarding their outcomes, not being effectively contrasted through the present study’s methodological approach; these were: A1-A2 and I1-I2. In addition, the reported findings seem to suggest that, amongst all measures, the high-frequency formants F3 and F4 were potentially the most inter-speaker discriminatory in identical twin pairs comparisons, whereas F1, F3, and F4 appeared to be the most discriminatory in comparisons involving NGRS. Finally, it was possible to verify that the overall F1-F4 formants’ discriminatory power appeared relatively higher for NGRS, with a total percentage of 33% differences among speakers, compared to identical twin pairs, for which a percentage of 18% differences was observed (i.e., 16% when neglecting marginally significant differences).

### Differences in the Bark scale

Regarding the comparisons intra-twin pairs using the Bark critical-band scale, some dissimilarities were observed compared to the to the Hertz scale results, all of which corresponded to the front vowel /i/ and mostly in higher formant frequencies (i.e., F2, F3, and F4). Four comparisons that were significant in Hertz have shown to be only marginally significant in the Bark scale (*p* ≤ 0.10/2): D1-D2 (F2); E1-E2 (F3); G1G2 (F4); J1-J2 (F4). Also, three significant comparisons in Hertz were found to be non-significant in Bark for the same vowel /i/: G1-G2 (F2); D1-D2 (F3); D1-D2 (F4). Such dissimilarities regarding the non-significant differences in Bark stand for only 6.5% of the total number of significant differences observed in Hertz. Apart from these observations, there seemed to be a noteworthy agreement regarding the comparison of statistical results across scales. Overall, 93.5% of differences observed in Hertz were also significant or marginally significant in Bark.

### The discriminatory power of phonetic vowels in BP

As in the analysis of mean formant frequencies, the speaker-discriminatory potential of individual vocalic segments in the present study was assessed while considering the number of cases in which the production varied significantly across speakers, yielding statistically significant differences. Such differences are displayed according to an articulatory phonetic criterion, namely the distinction amongst front, central, and back vowels.

It can be seen in Tables 1 and 2 that amongst the phonetic vowels of BP, the central vowel /a/ was the one displaying the highest number of significant differences in both identical twin pairs and NGRS groups, followed by the front vowels /i/, /ε/ and /e/, respectively. Furthermore, from the back vowels class, the vowel /u/ seemed to display the highest discriminatory power for NGRS, whereas for identical twins, both /ɔ/ and /u/ were found to portray a relatively low-discriminatory potential.

As can be seen in Table 1 for both F1/F2 and F3/F4 formant groups, front vowels seemed to be, in general, considerably more speaker-discriminatory than back vowels. In terms of vowel quality-related parameters in BP (F1-F2), only two identical twin pairs were contrasted through the analysis of the back articulatory dimension, whereas five twin pairs were effectively contrasted by the comparison of front vowels. When considering all formant frequencies (F1-F4), an average of nine significant differences for the former and three for the latter vowel category were observed. Notably, F2 average values from back vowels appeared to be the parameter displaying the lowest inter-speaker discriminatory potential concerning identical twin pairs.

Although in a relatively smaller proportion, a discrepancy in the discriminatory power for front and back vowels was also found for NGRS, as shown in Table 2 (see front and back mean values). Again, the mean F2 frequency for back vowels was the parameter displaying the lowest inter-speaker discriminatory power.

In Table 5, effect size estimates can be visualized as a function of vowel quality in comparisons carried out among all speakers. Regarding the combination of stressed and unstressed vowels, the front vowel /ε/ was the only vowel displaying only large effect sizes, suggesting higher differences in mean formant values among individuals for this vowel, as well as a greater explanatory power of the variable “speaker” on the observed differences. In summary, all reported differences concerning vowel quality ranged from moderate to large effect sizes, except for the back vowels /o/ and /u/, for which small magnitudes regarding F2 were found. This outcome suggests smaller differences in terms of F2 average values among speakers in such segments.

**Fig 1:**
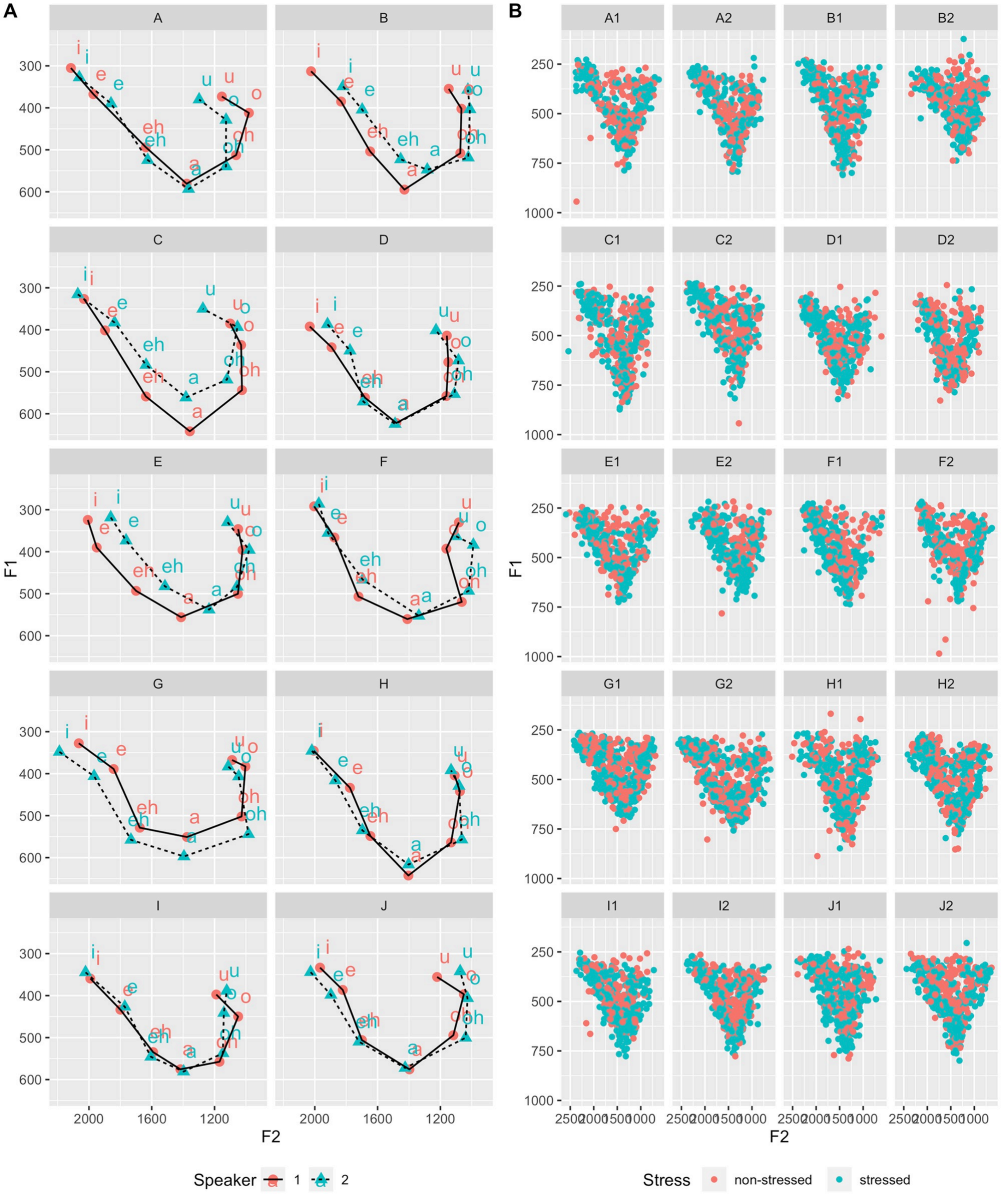
Two-dimensional vocalic space comparisons between identical twin pairs (A) and stressed and unstressed vowel data points by speaker (B).

**Fig 2:**
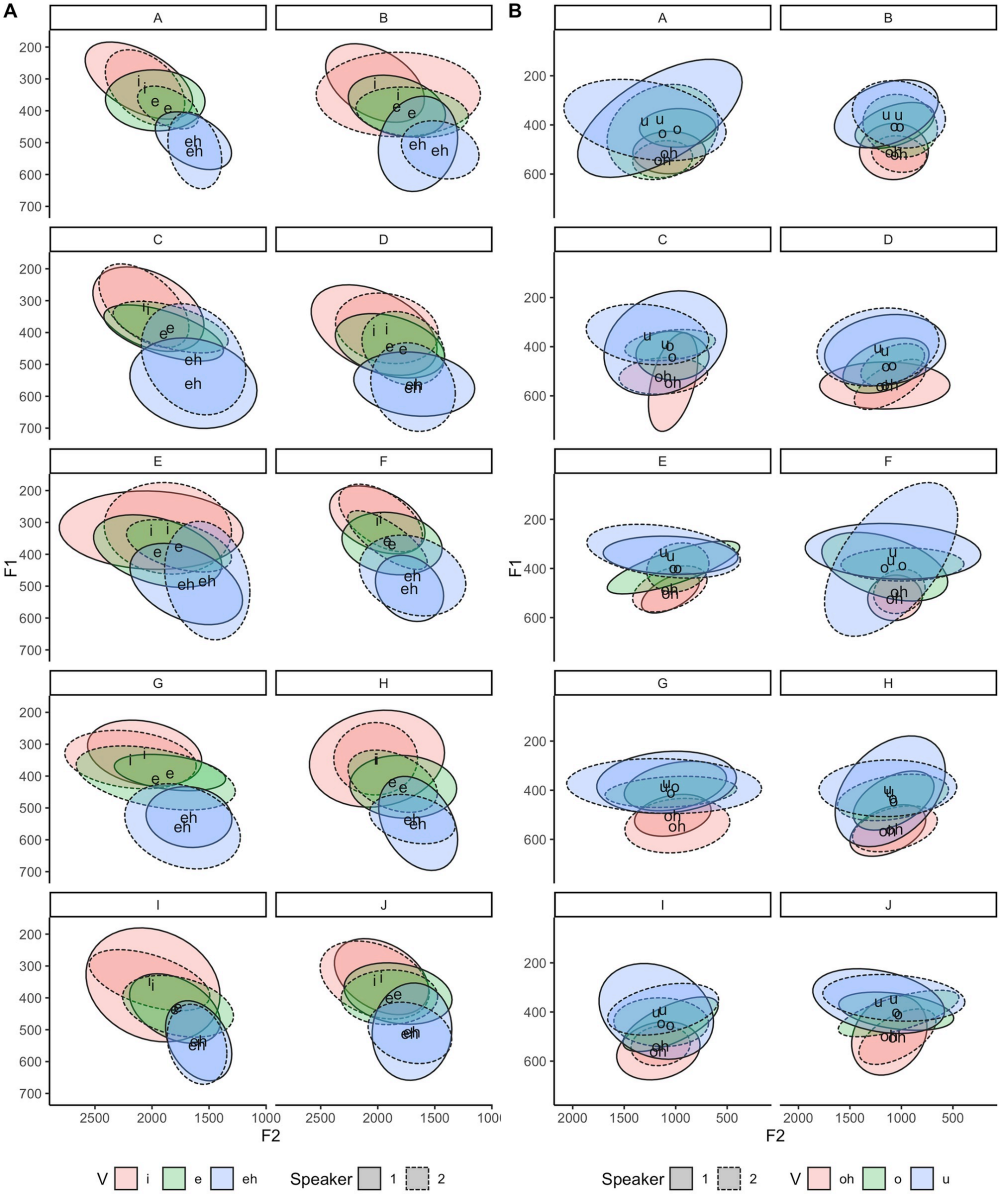
Front vowels (A) and back vowels (B) vocalic spaces’ means and confidence interval for intra-identical twin pairs comparisons.

Representations of the two-dimensional vocalic spaces of twin pairs are presented in Fig 1A. By visually inspecting the over-plots, it is possible to observe how closely related such individuals are in terms of their vocalic acoustic patterns, specifically in terms of their F1 and F2 mean values. Concerning the phonetic variability in the front/back articulatory dimensions, in Fig 2. diagrams representing the areas corresponding to a confidence level interval of 95% are displayed for each front and back vowel and each twin pair. In this Figure, the area inside the ellipses corresponds to 95% of observed data points, in which average values are expressed by the vowel letters (note that: /eh/ = /ε/, and /oh/ = /ɔ/).

In Fig 2 a higher degree of overlap between the ellipses for back vowels suggests closer phonetic-acoustic proximity for these vowels compared to the front ones. Furthermore, such phonetic-acoustic proximity was confirmed by the assessment of the Euclidean distances between neighboring vowels in the cardinal vowel space.

As shown in Table 6, which presents the intervocalic Euclidean distances and mean F1-F2 values between phonetic vowels, there was a tendency for front vowels to be considerably more dispersed when compared to the back ones. The Wilcoxon test revealed statistically significant differences for vowel height (F1) in the comparison between /u/ and /i/, /o/ and /e/ (*p* < 0.001) with the exception of /ɔ/ and /ε/ (*p* = 0.7). In this case, the back vowels /u/ and /o/ were considerably lower than the matched front vowels, resulting in a vocalic asymmetry.

**Table 6:** Intervocalic Euclidean distances and mean F1-F2 values between phonetic vowels.

Vowel class	Vowel pairs	Euclidean dist (Hz)	Euclidean dist (Bark)	F1 diff (Hz)	F2 diff (Hz)
Front	i—e	175	0.84	65	163
	e—ε	235	1.33	123	200
	i—ε*	409	2.18	188*	363*
Back	u—o	111	0.59	44	82
	o—ɔ	93	0.98	109	24
	u—ɔ*	164	1.42	153*	58*
Central	a—ε	273	1.27	61	266
	a—ɔ	317	1.67	59	312

Furthermore, the entire front and back vertical articulatory dimensions also seemed to exhibit a height discrepancy, in which the F1 mean difference in distance between the extreme front vowels /i/-/ε/ and the extreme back ones /u/-/ɔ/ was found significantly discrepant (*p* < 0.001). The comparison of the Euclidean distances between the cardinal vowels displayed in Table 6 also appeared to corroborate this discrepancy related to articulatory working space, especially when considering the extreme vowels in the front articulatory dimension /i/-/ε/ and back /u/-/ɔ/.

With regard to vowel formant variability, front vowels were found more variable for both F1 and F2, with mean standard-deviations of 94.8 Hz and 250.9 Hz, respectively, compared to back vowels, for which mean standard-deviations of 84.2 Hz and 227.1 Hz were observed, respectively. The reported variance differences were found statistically significant when performing the Fligner-Killeen test of homogeneity of variances, suggesting different variances for front and back vowels (F1: *chi*-*squared* = 61.731, *p*-*value* < 0.001; and F2: *chi*-*squared* = 75.129, *p*-*value* < 0.001).

### The lexical stress effect

Contrary to what was initially hypothesized concerning the stress effect on twins and NGRS’s formant frequencies, the outcomes reported in Tables 3 and 4 suggest the same trend in terms of phonetic-acoustic differences for both groups. In general, stressed vowels appeared to display a relatively higher discriminatory power in absolute numbers than the unstressed ones. Furthermore, all identical twin pairs that were contrasted through unstressed vowels were also discriminated by stressed vowels comparisons, except for one pair: D1-D2.

From a more comprehensive approach, by considering the combination of stressed and unstressed vowels, as presented in Tables 1 and 2, an even higher number of significant differences were found for both groups of speakers, which may suggest the combination of stressed and unstressed vowels as being more explanatory in terms of the differences observed amongst individuals.

**Fig 3:**
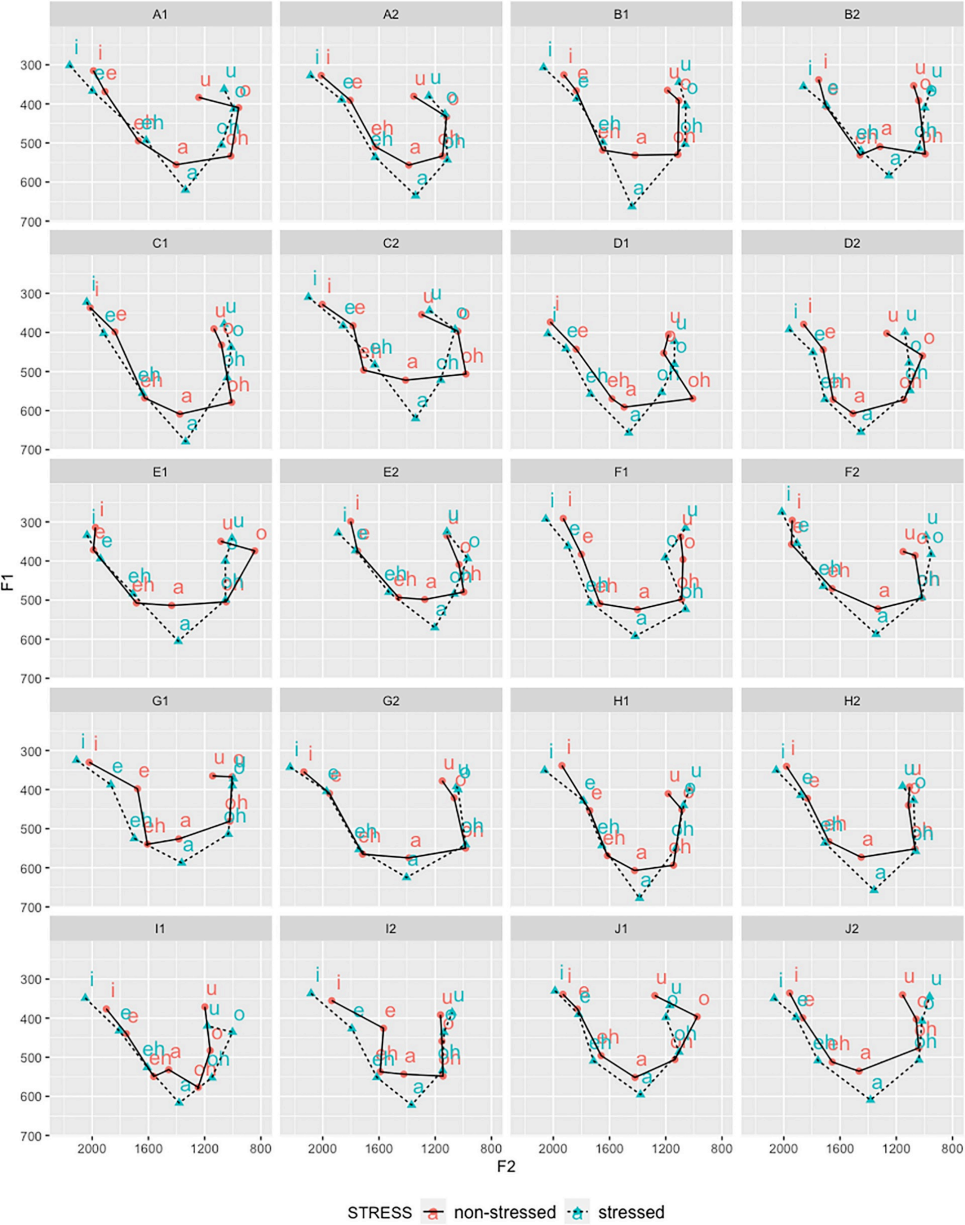
Effects of lexical stress in the two-dimensional vocalic space intra- and inter-speakers.

Through visual inspection of Fig 1B, which displays the data points corresponding to stressed and unstressed vowels by each speaker, it can be observed how their vowels are dispersed in the vocalic system. A higher concentration of stressed vowels in peripheral regions, as well as a more centralized dispersion of unstressed vowels, were suggested. This tendency was confirmed when mean F1 and F2 frequencies are plotted in the vocalic space. As can be visualized in Fig 3, unstressed vowels’ formant frequencies displayed a tendency to be more centralized in relation to stressed vowels, which seemed to be particularly the case for the central vowel /a/, resulting in a vertical reduction of the vocalic acoustic space.

When analyzing effect sizes as a function of lexical stress in Table 5, it is possible to observe magnitudes ranging from moderate to large in stressed vowels and small to large in unstressed vowels. Moreover, the F1 of stressed vowels was the only parameter displaying only large effect sizes. In general, in both stressed and unstressed conditions, effect sizes were relatively smaller for F2 than for F1. Finally, regarding individual vowels, the stressed vowels /a/, /i/, /ε/, and the unstressed vowels /e/ and /ɔ/, were the ones displaying only large effect sizes. That is three front vowels, one central and one back.

With regard to vowel formant variability, stressed vowels were found more variable than unstressed vowels for F1 and F2, with an observed standard deviation of 128.2 Hz and 403.7 Hz in stressed vowels, respectively, compared to 121.3 Hz and 355.3 Hz in unstressed vowels, respectively. Such difference was also statistically significant when performing the Fligner-Killeen test of homogeneity of variances, suggesting that, in fact, stressed and unstressed vowels display different variances (F1: *chi*-*squared* = 31.268, *p*-*value* < 0.001; and F2: *chi*-*squared* = 118.41, *p*-*value* < 0.001).

## Discussion

The present study proposed an analysis on the speaker-discriminatory power of vowel formant frequencies in comparisons of genetically-related, namely identical twin pairs, and unrelated speakers. The effect of lexical stress regarding the formant’s discriminatory potential was also considered. The main findings are discussed in the following sections.

### F1-F4 discriminatory power in identical twins and NGRS

As observed in the present study, for both identical twin pairs and NGRS, high-formant frequencies appeared to be more speaker discriminatory in comparison to lower formant frequencies. This finding is in broad agreement with trends reported in the literature regarding experiments with identical twins in both controlled and uncontrolled speech [1, 2, 8, 30]; as well as with non-genetically related individuals [13].

Given the control of the linguistic component on the limits of variation for vowel quality-related formants, as justified on account of articulatory reasons [26], and the superposition of physiological and linguistic components on identical twin pairs, fewer significant differences for F1 and F2 compared to higher formants may be presumed. For NGRS, however, the impact of the linguistic component, as in the case of the shared dialect, seems to apply, especially for F2. Furthermore, this outcome may be related to a lower variation for F2 observed in back vowels, as shown in Table 2. It is worth mentioning that the F2 dimension is importantly related to vowel place of articulation, serving as an acoustic indicator of the constriction position in vowel production [26]. In this sense, the comparisons within the NGRS group may suggest that differences for F1 appear to be more tolerated than differences for F2. Notwithstanding, this trend can not be generalized for identical twin pairs, where a slightly higher discriminatory power was observed for F2 than for F1, especially for the front vowels, which demands further investigation.

As for higher formants, such as F3 and F4, lower linguistic constraints may be presumed for both groups of speakers, namely identical twins and NGRS. Such observation invites the hypothesis of high formants’ resonance variations (i.e., associated with vocal tract configurations and individual phonatory settings) as possibly more speaker-discriminatory, as justified by the observation of such frequencies as less dependent on the phonetic quality of sounds, which appears to be particularly the case for F4. In that regard, as observed in the experiment conducted by [28], the fourth formant frequency (F4) seemed to be mainly sensitive to laryngeal cavity changes while insensitive to changes in the upper part of the vocal tract.

Two main widely acknowledged factors may be related to the level of phonetic variation observed in identical twins and NGRS, namely physiological and linguistic factors. The implied relation between these two components has already been addressed by [45] when highlighting that physically-related acoustic dimensions, such as fundamental frequency and formant frequencies, are equally exploited by languages and are therefore conflated with linguistic information. In that sense, while the physiological component may establish the limits of physical variation, the linguistic component is responsible for keeping parameters constant, restraining the variation allowed by the linguistic system. Either the higher or lower superposition of these two dimensions may possibly account for the differences observed between twin pairs and NGRS in the present study. Different levels of superposition of such dimensions, even between identical twins, may be observed, given the fact that some identical twin pairs were found to be more similar than others within the formant frequency domain.

The verification that a lower discrepancy between the discriminatory potential of F1 and F2 was verified for identical twin pairs (4%) when compared to NGRS (16.5%) is noteworthy, as revealed by the comparison of the first two rows in Tables 1 and 2. Such an outcome may be potentially interpreted as the result of a comparable influence of linguistic and structural components on identical twins’ linguistic output, yielding a higher similarity in their productions. The same does not apply, however, to all speakers. The F1 and F2 dimensions seemed to diverge to a greater extent for NGRS, for whom a non-analogous influence of linguistic/environmental and structural factors are implied.

Notably, studies carried out with identical twins and non-identical twin speakers, as in the case of siblings or unrelated subjects, seem to corroborate this greater phonetic similarity observed concerning identical twin pairs, providing evidence that, saved some exceptions, a higher phonetic convergence is present in such individuals, as verified through the analysis of electromagnetic articulography [31], acoustic speech [8, 33, 37] and voice analysis [7, 33, 46].

As observed by [37], given the extent of genetic influences on peripheral structures involved in speech production, such as the vocal tract and the larynx structures, it is expected that higher levels of physical similarity may influence speech characteristics of identical twins. Another critical variable to be considered refers to the implications of the shared linguistic environment on speech behavior. It can be hypothesized that the greater the influence of the linguistic component, the lower is the variability expected regarding a particular physical measure. However, determining the contribution of these two components in the shaping of speech patterns remains a challenge for phonetic research.

Considering that in [35], identical twins raised apart exhibited the same degree of similarities in their vocalic transitions as identical twins raised together did, suggest a greater influence of physiology over learning regarding dynamical features. Moreover, in [31], MZ twins that were more frequently in contact with each other displayed comparable levels of similarities comparable than to those that were less frequently in contact. However, it is worth noting that all twins in the latter study were brought up and lived together as teenagers, which does not allow one to question the environmental influence over the twins, as in the case of the first study.

Conversely, evidence pointing to substantial influence of environmental factors on speech patterns have been presented by [34], while verifying that not only MZ twin pairs but also other siblings were able to deteriorate the performance of a forensic-comparison system based on vowel formant trajectories. According to the authors, factors such as learned variation, individual choice, and the attitude towards one’s own sibling seem to play an important role in speech production, and can possibly explain convergences in non-identical individuals.

Finally, the mere fact that identical twins have shown to vary substantially in terms of F3 and F4 in the present study suggests that these formants may not be solely dependent on fixed structural features and are as well influenced by dynamical aspects in speech production. Differences concerning these formants have been consistently and systematically reported by other studies in comparisons involving identical twin pairs [1, 2, 8, 30].

### Identical twin pairs comparisons in Bark and Hertz scales

The primary motivation for applying the Bark critical-band scale in the twins’ comparisons was to verify whether differences would also be potentially significant when following a psychoacoustic criterion. This verification is especially relevant since variations in anatomic and physiological components involved in speech production could perhaps not be enough to account for all differences observed in identical twin pairs. The verification of convergence in the results between the two scales could imply that the twin pairs may, potentially, be able to perceive such differences, inviting the variable “choice” as one possible explanatory component. Notwithstanding, future studies are needed to corroborate this assumption while also estimating the magnitude of the differences observed.

According to [47], it is possible to categorize speaker-discriminatory variables, or in the terms used by the author “speaker-diagnostic”, through two basic distinctions: *organic* versus *acquired/learned*, and within the latter: *individual* versus *group*. As described in [48], the first category has to do with the structure of the vocal organs of speakers and their vocal tract dimensions. Even though such aspects are fundamentally determined by *organic* factors, they are susceptible to suffer modification by learning, as pointed out by the authors.

Concerning the second category—*acquired* or *learned* characteristics—the distinction between *group* versus *individual* is necessary. According to [48], group characteristics are related to social, regional, and cultural conditions, while individual or idiosyncratic features refer to individual variation within a particular group, expressed by patterns that are not predicted from a group perspective. The variable “choice” is inserted within this domain.

In regards to speech production, as mentioned in [1], “choice” has to do with the selection or adoption of articulatory patterns from available role models or alternative articulatory strategies to satisfy the phonological requirements of a target segment. The level of variation allowed for alternative realizations appears, however, to be determined and regulated by the phonological system, as commented furthermore.

Whether “choice” is a conscious or an unconscious process, it likely may require some degree of perceptual processing. An alternative realization that cannot be perceived by the speaker or that is not linguistically salient may be unlikely to persist. In that sense, some level of auditory feedback might be required.

Moreover, the variable “choice” has been widely considered as one of the probable explanations for variations observed between very similar speakers, as in the case of identical twin pairs who had grown up and lived together [1, 4, 34, 35]. The possible implications of choice as an attempt to establish an individual linguistic identity, especially in contexts where this identity competes or is challenged by another, remains to be explored.

### The speaker-discriminatory potential of vowels in BP

Differences within identical twin pairs and NGRS as a function of vowel quality in BP pointed to a similar trend for both groups. This observation is in line with the assumption that differences between groups would be mostly explained in terms of mean formant frequency analysis rather than for individual vowel quality, given the fact that all individuals in the present study spoke the same dialect.

From a qualitative viewpoint, the central vowel /a/ appeared to be the most discriminatory segment in both groups, followed by front vowels. Along with the back vowel /u/, the central and front vowels were also the most frequent ones, as previously mentioned. Regarding identical twin pairs, the following vowel discriminatory power was suggested, based on the frequency of statistical significant differences (from higher to lower): /a/, /i/, /ε/, /e/, /oh/ = /u/, /o/, respectively. As for NGRS, the overall vowel discriminatory power suggested was (from higher to lower): /a/, /i/, /ε/, /e/, /u/, /oh/, /o/, respectively.

The observation that the best performing vowels were also the most recurrent ones, as in the case of /a/ and /i/, may present some statistical implications. It is common knowledge that larger data samples tend to reflect more reliable estimates and are more likely to reveal significant differences in lower alpha values [8]. Notwithstanding, the observation of a higher frequency of occurrence regarding some specific tokens may be considered itself a justification for electing such vowels as viable for the forensic speaker comparison task in contexts where no substantial data is available for analysis. It is worth mentioning that “disponibility” is a crucial factor that comprises one of the main criteria for the parameter selection in the FSC domain. Such a factor refers to the frequency of occurrence of a given element or phenomenon in speech [45].

Despite the difference observed concerning the frequency of occurrence of front and back vowels in the present study, there is reported evidence in the literature of front vowels as being more speaker-specific in Australian English, with F2 of back vowels being considerably homogenous in comparisons involving similar-sounding speakers [3]. Also, in [4], differences in vowel realization were found where some speakers had more fronted vowels than their twin pairs during spontaneous conversational speech.

According to [49], patterns of vowel variability appear to be conditioned differently by contextual and non-contextual factors. In their research with Catalan dialects, front vowels (mostly /i/) were found to be very resistant to context-dependent effects, whereas back vowels varied more along the F2 dimension. Regarding non-contextual variation, i.e., idiosyncratic variation, the opposite trend was observed. Overall, there was more F1 variability for low vs. high vowels and more F2 variability for front vs. back vowels. As pointed out by the authors, “random” variability depends inversely on the precision involved in achieving the articulatory target for a given vowel. In the same direction, while analyzing F2 and F3 transitions in American English, [50] observed that front vowels were less contextually variable than back vowels.

Overall, the present study’s findings appear to be in line with what was reported by [3, 4, 49] while suggesting a higher individual variability for front vowels. Regarding the present study’s data, it can be assumed that contextual effects have a comparable influence on the patterns observed across individuals, given the representatives of the material analyzed, which includes a high number of vowel tokens produced in several different phonetic contexts, which is believed to yield a contextual effect minimization. From this viewpoint, the differences observed are more likely to be mostly related to idiosyncratic phonetic patterns rather than contextual ones.

One possible explanation regarding the differences observed for acoustic distances between cardinal vowels in this dialect of BP, and perhaps for the higher variability observed for the central and front vowels, is that the perceptual mechanism “knows” that such vowels are usually more variable than the back ones, perhaps due to articulatory working space constraints, performing a perceptual compensation for such discrepancy [25]. From an articulatory viewpoint, as referred by [3], speakers may be using different articulatory strategies for front vowels aiming to produce phonologically equivalent vowels. Notwithstanding, this would also imply differences in the articulatory feedback for front, central, and back vowels in BP, as more variation appears to be tolerated for the first and second vowel groups. Conversely, a higher articulatory precision seems to be required for back vowels in BP, given the noticed lower variation for this vowel class, particularly concerning the articulatory horizontal dimension.

Given the fact that the vocalic system of BP is considered relatively symmetric, with seven peripheral oral vowels in a stressed position and the same number of front and back vowels, a homogeneous vowel dispersion could be expected, as expressed by an even spacing of neighboring vowels in the acoustic space. In contrast, a discrepancy in terms of acoustic distances between the entire front and back articulatory dimensions was observed, besides the already reported height asymmetry between these two phonological categories [15]. A plausible question concerns whether this acoustic space discrepancy would be related to the lower levels of variation observed for back vowels, given that increased proximity between vowels due to alternative articulatory realizations could imply perceptual difficulties. Another support to an articulatory working space discrepancy may be provided by the fact that in asymmetric phonetic inventories in languages of the world, the number of front vowels is likely to be greater than the number of back vowels, three times more likely in primary systems and two times more in secondary systems [51, 52].

As mentioned previously, the central vowel /a/ was the vowel displaying the highest inter-speaker discrimination score from the set of vowels analyzed. In terms of vocalic dispersion, this is the only central vowel in the BP system and also the one displaying the highest acoustic distances from its neighbors in the current analysis. The combination of these two factors may place this vowel in a favorable position for a higher phonetic-acoustic variability, as observed in the present study. Notwithstanding, the fact that the vowel /i/ appeared to be the most discriminatory front vowel, even though it was not the one displaying the highest Euclidean distance from its neighbor, may indicate that greater acoustic distance between vowels is not the only factor accounting for the variation in vowel production. In this sense, the concept of “sufficient contrast” [53], meaning the phonetic distance between different vowels may also play a role.

When assessing two performance constraints, namely articulatory simplification, and perceptual distinctiveness, [54] states that vowels in vocalic systems of languages in the world seem to have evolved more than anything in response to a distinctiveness demand. In this regard, according to his general theory of vowel adaptive dispersion, vowels tend to evolve to provide both sound and feel sufficiently different from each other. This is in line with the claims of [52] when highlighting that vowel systems should optimize auditory distances in order to enhance contrast and provide as much information as possible about articulatory gestures. According to the authors, if it is true that the interactions between speakers and listeners are responsible for shaping phonological inventories, then, as a result, phonological inventories may provide information about the speaker-listener interaction mechanism.

As was observed in the present study, if it is the case that acoustic distances between neighboring vowels—which have direct implications for perceptual distinctiveness—are in part related to their level of phonetic variation, then it could be justified why the central /a/ vowel in BP seems to be the most inter-speaker discriminatory segment. The acoustic specification regarding this vowel and its position in the vocalic system would contribute to the higher level of variation observed. Considering that /a/ is the only central vowel in BP vocalic system, it may be suggested as less likely to be perceptually confused with other vowels during alternative or imprecise realizations. Notwithstanding, future acoustic and perceptual research are required in order to validate this hypothesis.

Finally, it is worth mentioning that vocalic systems with larger or smaller vowel inventories and different vocalic dispersions may display different variation patterns, since phonetic content is regarded as dependent on system or inventory size [54]. In [55], while referring to consonant systems, the author points out that in small systems, demands for perceptual distinctiveness tend to be less significant than in larger systems. In addition, complex articulatory patterns seem to be required due to a higher intra-system demand for contrast. This trend also appears to apply to vocalic systems, as observed by [51]. By analyzing the structure of 317 primary and 121 secondary systems, as an attempt to identify major trends in vowel inventories, the authors observed that, in general, vowel systems first tend to exploit a ‘‘primary’’ system of sounds. However, when systems are found to exceed the inventory size of nine vowels, there is a clear tendency for exploiting at least one new dimension, the so-called ‘‘secondary’’ systems, often represented by secondary articulations or duration contrasts.

As for the before-mentioned reasons, forensic-phonetic studies should appraise the language-dependent nature of phonetic variation, especially when outcomes obtained with different language systems are compared.

### The lexical stress effect

The analysis of vowel formants’ discriminatory power as a function of the lexical stress component in the current study was grounded on the fact that different data distributions may exist concerning distinct vowel groups. In that regard, different vowel distributions could yield distinct patterns concerning vowel formants’ discriminatory power.

In the present study, the lexical stress effects on vowel formants revealed similar trends for comparisons involving both identical twin pairs and NGRS. The comparisons carried by the combination of stressed and unstressed vowels appeared to be the most explanatory measure in terms of differences observed among speakers, as expressed by the relatively higher number of significant differences.

When assessing the stress component separately, by comparing speakers for stressed and unstressed vowels independently, it was found that formant measures obtained through the assessment of stressed vowels seemed more discriminatory than those extracted from unstressed vowels. This finding seems to partially agree with the results reported by [8], in which identical twin pairs were found more acoustically similar in the production of unstressed vowels and more distinct for the realization of stressed vowels. According to [8], one possible explanation for such an outcome is the assumption of anatomic and physiological aspects as having a more substantial impact on auditorily less salient parameters. The author also observed that MZ twins were more similar in the production of unstressed syllables when compared to DZ twins. Nonetheless, the present study does not corroborate this finding, as the same trend regarding the stress effect was observed for both similar and non-similar group of speakers.

The verification of stressed vowels as being relatively more dispersed in the BP vowel space than unstressed vowels may be considered one plausible factor partially accounting for the discriminatory patterns observed. The assumption of stressed vowels as tending to be more clearly articulated, less reduced, could imply such segments as more acoustically contrasting. In this regard, it may be hypothesized that the presence of the stress component may reflect a better portrayal of individual articulatory adjustments, and consequently, of inter-speaker differences. Conversely, unstressed vowels are suggested as more susceptible to effects such as vocalic reduction, which according to [56], may be the consequence of a general principle, namely the realization of the energy downstep necessary for the distinction between stressed–unstressed sequences.

As the results of [24] suggest, although tempo and stress may not have a major influence on the distances of individual vowels from the neutral point, the size of the vowel space overall appears to be susceptible to the effect of these variables. In the study conducted by the researcher, there was a general tendency for larger vowel spaces for the slow stressed condition and smaller for the fast unstressed condition, which may suggest a relationship between precision in speech production and the overall configuration of the vowel space.

Another relevant aspect regards the acoustic salience of segments. Because stressed vowels in BP are referred to as more salient from a perceptual point of view, presenting higher duration, higher *f*0 standard-deviation, and higher spectral emphasis [57], such segments may be suggested as being potentially more targeted by speakers when implementing alternative realizations. In that regard, the literature signals the critical role of perception when speakers attempt to implement different speech patterns, as in the case of categorical production of pitch variations in imitation tasks [58].

Duration has been considered to be the most reliable exponent of stress across different languages [59]. As such, further analysis should also explore the extent to which the higher duration commonly reported for stressed vowels might be related to a higher level of phonetic variation among speakers, as the possible result of an increase in the time-span for articulatory differences to emerge.

The present study also found evidence suggesting a vertical reduction of the vowel space in an unstressed condition for all speakers and a horizontal vowel space reduction in some particular cases. This observation is in broad agreement with what was reported by [23] concerning the non-peripheral status of /a/ in a post-stressed position in BP. The acoustic outcomes reported by the author revealed the intermediate acoustic nature of the final unstressed /a/ regarding its stressed production and the center point in the vocalic space (centroid), which also resulted in a vowel space vertical reduction. This tendency for vowel centralization in unstressed vowels has also been reported for other languages, as in the case of the Spanish vowels /a/ and /o/ [22], and the vowels /a/ and /e/ in Hebrew [21], as previously mentioned.

Furthermore, one trivial factor may also be related to the discriminatory pattern observed as a function of lexical stress, namely the number of observations for each variable, which is unquestionably a crucial aspect in any statistical inference analysis. In that regard, from the 9,446 vowel points analyzed in the present study, 5,487 (58%) were classified as stressed, while 3,959 (42%) as unstressed, a difference of 16% in the number of data points between the two categories. As such, given the impact of n-size on the statistical strength, a higher number of statistical differences could be presumed for the stressed condition. The same reasoning applies to the combined stressed and unstressed condition, where an even higher number of data points is present, and a greater number of statistical differences were observed.

A statistical estimate that is able to compensate for such data discrepancy is the analysis of the effect sizes. As mentioned by [44], while p-values are largely influenced by sample size and more likely to be significant when the sample size is large and less likely if the sample is small, effect size estimates, in contrast, are not sensitive to it, providing a standard metric to compare the direction and strength of the relationship between variables. As observed in this study (see Table 5), stressed vowels tended to display large effect sizes in a higher number of vowels when compared to unstressed vowels. Notwithstanding, when comparing the mean inter-speakers differences in effect size’s magnitude for both vowel classes, a small difference was observed, which may be due to a considerably higher quantitative effect size estimation for most unstressed vowels, particularly regarding F1. The reason for such a difference remains still unclear.

### Implications for forensic-speaker comparisons

The implications of the present study’s outcomes on the forensic speaker comparison practice may be qualified as two-fold, as it comprises both a theoretical and a practical demand. The first demand regards the necessity of better understanding the limits of phonetic variation among speakers, including those who display a considerably high level of superposition, and identifying possibly explanatory factors accounting for their speech patterns.

The second demand regards a common goal in forensic phonetics research, namely the identification of relevant and robust parameters for the forensic speaker comparison practice. Here the focus resided on vowel formant frequencies, particularly inter-speaker discriminatory in comparisons involving genetically-related and NGRS. Most importantly, such parameters were assessed through an ecologically-valid material comprised of spontaneous speech samples. As [3] points out, if inter-speaker differences can be observed in vowel tokens that are not strictly controlled for phonetic context, the potential number of tokens available for forensic analysis is increased. Moreover, the fact that differences between very similar speakers could be found when an “economic” measurement approach, involving an estimate over a single temporal interval in the vowel’s midpoint, is highly relevant from a forensic phonetic perspective.

Regarding the requirements on the selection of forensic phonetic parameters, [45] defines some relevant criteria, among which are “disponibility” and “measurability”. The first aspect refers to the frequency of occurrence of a given element, being of particular relevance in real forensic contexts, in which large questioned samples are seldomly available for the experts to base their judgments on. Such aspects are related to the forensic data’s representativeness, as remarked by [25]. In this sense, the strength of the evidence is dependent on how well the questioned and known suspect samples reflect their respective sources, which is consequently dependent on the amount and the quality of the data available.

In the present study, the observation of some specific vowels as being relatively more frequent, aligned with their apparent higher discriminatory potential, as in the case of the central vowel /a/ and front vowels, may indicate such units as ideal for the forensic speaker application in BP, particularly in contexts involving a shortage of data. Notwithstanding, the discriminatory potential of all vowels must be acknowledged in forensic casework whenever possible.

Notably, some crucial limitations may be identified concerning the application of vowel formant analysis in forensic contexts, as in the analysis of telephone-transmitted recordings. The effects of the telephone band-pass on the acoustic signal is commonly referred to as “the telephone effect” (cf. [60]), mainly characterized by the suppression of higher formant frequencies due to the lower cut-off slope of the telephone band-pass, and the tendency of lower formants to be shifted. As observed by [60] in an experiment with German speakers, low formants of vowels produced by males and females tended to move upwards in telephone-transmitted samples compared to direct recordings, resulting in erroneous measurements. This effect has also been verified by [61] for male speakers in BP, in which the increase of the F1 values in the mobile phone situation caused a global downward displacement of the vowel space. In contrast, the decrease of the F2 values for the front vowels and the increase of this formant’s values for back vowels resulted in a vowel space reduction.

Notwithstanding, there may be situations in which the analysis of vowel formants in telephone speech might be relevant, that is, when both questioned and reference materials are telephone-transmitted. As pointed out by [60], as long as both questioned and reference material in a forensic case were recorded via telephone, there would not be serious implications, assuming that different telephone channels do not differ substantially in terms of their practical effects.

Finally, as pointed out by [13], voice communication by other means than telephone has become increasingly common, as in the case of cross-platform messaging apps (e.g., WhatsApp and Telegram), allowing users to exchange voice messages. Such technological advances have introduced the possibility of applying higher formant frequencies in forensic speaker comparisons, such as F3 and F4, as well as other acoustic measures (e.g., voice quality parameters), which certainly demands more experimental work to be done.

Lastly, it must be acknowledged that studies with vowel formants are also relevant in the domain of speech technology, providing the experimental background for speaker recognition system’s enhancement.

## Conclusion

This study on the speaker-discriminatory power of vowel formant frequencies found evidence suggesting high-formant frequencies, namely F3 and F4, as potentially more speaker discriminatory than low-formant frequencies, as verified by the qualitative comparison of statistically significant differences across speakers as well as the comparison of effect sizes.

The comparisons between groups revealed different phonetic variation levels for identical twin pairs and NGRS, with identical twin pairs appearing to be relatively more similar than NGRS regarding their outcomes. Nevertheless, the results also suggest that these individuals are not phonetically identical and can avail of the same articulatory freedom allowed by the shared phonological system, as reported by previous studies. Overall, eight pairs of identical twins were efficiently contrasted by the comparison of their vowel formant frequencies. Notwithstanding, two identical twin pairs remained undifferentiated through the present study’s approach.

Regarding the inter-speaker discriminatory potential as a function of vowel quality, evidence was found suggesting the central vowel /a/ and front vowels as the most speaker-discriminatory segments. These segments also seemed to display higher Euclidean distances from their neighbors, which may invite the hypothesis of a probable relationship between vowel acoustic dispersion and the level of phonetic variation allowed by the phonological system.

Furthermore, even though stressed vowels appeared as more speaker-discriminatory than unstressed vowels, the combination of both vowel classes seemed to be more explanatory in terms of the inter-speaker differences observed. Nonetheless, this outcome can not be completely detached from the fact that a higher number of data points was involved in the combined stressed and unstressed condition.

Finally, further research is encouraged to assess the level of consistency regarding the patterns observed here, including the analysis of different speaking styles, such as interview vs. spontaneous dialogue.

## Supporting information

S1 Data (CSV)

## References

[1] Nolan F, Oh T. Identical twins, different voices. International Journal of Speech, Language and the Law. 1996;3(1):39–49. 10.1558/ijsll.v3i1.39

[2] Loakes D. A forensic phonetic investigation into the speech patterns of identical and non-identical twins. In: 15th International Congress of Phonetic Sciences (ICPhS-15). vol. 15; 2003. p. 691–694. Available from: https://www.internationalphoneticassociation.org/icphs-proceedings/ICPhS2003/p15_0691.html.

[3] Loakes D. Front Vowels as Speaker-Specific: Some Evidence from Australian English. In: Proceedings of the 10th Australian International Conference on Speech Science & Technology. January 2004; 2004. p. 289–294.

[4] Loakes D. A forensic phonetic investigation into the speech patterns of identical and non-identical twins. International Journal of Speech, Language and the Law. 2008;15(1):97–100. 10.1558/ijsll.v15i1.97

[5] Loakes D, McDougall K. Individual variation in the frication of voiceless plosives in Australian English: A study of twins’ speech. Australian Journal of Linguistics. 2010;30(2):155–181. 10.1080/07268601003678601

[6] Fernández ESS. Glottal source parameters for forensic voice comparison: An approach to voice quality in twins’ voices. In: International Association for Forensic Phonetics and Acoustics Annual Conference; 2012.

[7] San Segundo E, Tsanas A, Gómez-Vilda P. Euclidean distances as measures of speaker similarity including identical twin pairs: a forensic investigation using source and filter voice characteristics. Forensic Science International. 2017;270:25–38. 10.1016/j.forsciint.2016.11.020 27912151 PMC5698260

[8] Weirich M. The influence of NATURE and NURTURE on speaker-specific parameters in twins’ speech: Acoustics, articulation and perception. International Journal of Speech, Language and the Law. 2012;19(1):119–122. 10.1558/ijsll.v19i1.119

[9] Vogel F, Motulsky A. History and Development of Human Cytogenetics Human Genetics Problems and Approaches Springer-Verlag, Berlin Heidelberg New York Tokyo 1986; p. 20–24.

[10] Beck JM. Organic variation of the vocal apparatus The handbook of phonetic sciences. 1997; p. 256–297.

[11] Thompson PM, Cannon TD, Narr KL, Van Erp T, Poutanen VP, Huttunen M, et al Genetic influences on brain structure. Nature neuroscience. 2001;4(12):1253–1258. 10.1038/nn758 11694885

[12] Gold E, French P. International practices in forensic speaker comparison. International Journal of Speech, Language and the Law. 2011;18(2):293–307. 10.1558/ijsll.v18i2.293

[13] Cao H, Dellwo V. The role of the first five formants in three vowels of mandarin for forensic voice analysis. International Congress of Phonetic Sciences. 2019; p. 617–621. 10.5167/uzh-177494.

[14] Ladefoged P, Broadbent DE. Information conveyed by vowels. The Journal of the acoustical society of America. 1957;29(1):98–104. 10.1121/1.1908694 2525139

[15] Escudero P, Boersma P, Rauber AS, Bion RAH. A cross-dialect acoustic description of vowels: Brazilian and European Portuguese. The Journal of the Acoustical Society of America. 2009;126(3):1379–1393. 10.1121/1.3180321 19739752

[16] Behlau M, Pontes PAL, Gananca M, Tosi O. Spectrographic analysis of vowels formants in Brazilian Portuguese. ACTA AWHO. 1988;7:74–85.

[17] Kent RD, Vorperian HK. Static measurements of vowel formant frequencies and bandwidths: A review. Journal of Communication Disorders. 2018;74(June):74–97. 10.1016/j.jcomdis.2018.05.004 29891085 PMC6002811

[18] Traunmüller H. Articulatory and perceptual factors controlling the age- and sex-conditioned variability in formant frequencies of vowels. Speech Communication. 1984;3(1):49–61. 10.1016/0167-6393(84)90008-6

[19] Tsao YC, Weismer G, Iqbal K. The effect of intertalker speech rate variation on acoustic vowel space. The Journal of the Acoustical Society of America. 2006;119(2):1074 10.1121/1.2149774 16521769

[20] Harmegnies B, Poch-Olivé D. Formants frequencies variability in French vowels under the effect of various speaking styles. Le Journal de Physique IV. 1994;4(C5):C5–509. 10.1051/jp4:19945108

[21] Silber-Varod V, Khorshidi N, Levi L, Amir N. The influence of lexical stress on formant values in spontaneous Hebrew speech. The 19th International Congress of Phonetic Sciences. 2019; p. 3538–3542.

[22] Santiago F, Mairano P. The role of lexical stress on vowel duration and vowel space in two varieties of Spanish. In: Proc. 9th International Conference on Speech Prosody 2018; 2018. p. 453–457.

[23] Barbosa P. Do grau de não perifericidade da vogal/a/pós-tônica final. Revista Diadorim. 2012;12:91–107.

[24] Fourakis M. Tempo, stress, and vowel reduction in American English. The Journal of the Acoustical society of America. 1991;90(4):1816–1827. 10.1121/1.401662 1960277

[25] Rose P. Forensic Speaker Identification. vol. 3. Tayler & Francis; 2002. Available from: http://repositorio.unan.edu.ni/2986/1/5624.pdf.

[26] Stevens KN, House AS. Development of a quantitative description of vowel articulation. The Journal of the Acoustical Society of America. 1955;27(3):484–493. 10.1121/1.1907943

[27] Sundberg J. Ciência da voz: fatos sobre a voz na fala e no canto. Editora da Universidade de São Paulo; 2015.

[28] Takemoto H, Adachi S, Kitamura T, Mokhtari P, Honda K. Acoustic roles of the laryngeal cavity in vocal tract resonance. The Journal of the Acoustical Society of America. 2006;120(4):2228–2238. 10.1121/1.2261270 17069318

[29] Takemoto H, Mokhtari P. Acoustic analysis of the vocal tract during vowel production by finite-difference time-domain method. The Journal of the Acoustical Society of America. 2010;128(6):3724–3738. 10.1121/1.3502470 21218904

[30] Weirich M. Articulatory and acoustic inter-speaker variability in the production of German vowels. Universitätsbibliothek Johann Christian Senckenberg; 2010.

[31] Weirich M, Lancia L, Brunner J. Inter-speaker articulatory variability during vowel-consonant-vowel sequences in twins and unrelated speakers. The Journal of the Acoustical Society of America. 2013;134(5):3766–3780. 10.1121/1.4822480 24180787

[32] Weirich M, Simpson AP. Differences in acoustic vowel space and the perception of speech tempo. Journal of Phonetics. 2014;43(1):1–10. 10.1016/j.wocn.2014.01.001

[33] San Segundo E. Forensic speaker comparison of Spanish twins and non-twin siblings: A phonetic-acoustic analysis of formant trajectories in vocalic sequences, glottal source parameters and cepstral characteristics; 2014.

[34] San Segundo E, Yang J. Formant dynamics of Spanish vocalic sequences in related speakers: A forensic-voice-comparison investigation. Journal of Phonetics. 2019;75:1–26. 10.1016/j.wocn.2019.04.001

[35] Zuo D, Mok PPK. Formant dynamics of bilingual identical twins. Journal of Phonetics. 2015;52:1–12. 10.1016/j.wocn.2015.03.003

[36] Figueiredo RMd. Identificação de falantes: aspectos teóricos e metodológicos; 1994. Available from: http://www.repositorio.unicamp.br/handle/REPOSIP/270642.

[37] Whiteside SP, Rixon E. Speech characteristics of monozygotic twins and a same-sex sibling: An acoustic case study of coarticulation patterns in read speech. Phonetica. 2003;60(4):273–297. 10.1159/000076377 15004495

[38] Boersma P, Weenink D. Praat: doing phonetics by computer [Computer program]. Version 6.0. 37. URL http://www.praat.org/ Retrieved March. 2018;14.

[39] Zwicker E. Subdivision of the audible frequency range into critical bands (Frequenzgruppen). The Journal of the Acoustical Society of America. 1961;33(2):248–248. 10.1121/1.1908630

[40] Traunmüller H. Analytical expressions for the tonotopic sensory scale. The Journal of the Acoustical Society of America. 1990;88(1):97–100. 10.1121/1.399849

[41] Zwicker E, Fastl H. Psychoacoustics: Facts and models. vol. 22 Springer Science & Business Media; 2013.

[42] Tomczak M, Tomczak E. The need to report effect size estimates revisited. An overview of some recommended measures of effect size. TRENDS in Sport Sciences. 2014; p. 19–25.

[43] Fritz CO, Morris PE, Richler JJ. Effect size estimates: current use, calculations, and interpretation. Journal of experimental psychology: General. 2012;141(1):2 10.1037/a0024338 21823805

[44] Berben L, Sereika SM, Engberg S. Effect size estimation: methods and examples. International journal of nursing studies. 2012;49(8):1039–1047. 10.1016/j.ijnurstu.2012.01.015 22377339

[45] Nolan F. The phonetic bases of speaker recognition. Cambridge University Press; 1983.

[46] San Segundo E, Gómez Vilda P. Matching twin and non-twin siblings from phonation characteristics. VII Jornadas de Reconocimiento Biométrico de Personas. 2013; p. 10–17.

[47] Ladefoged P. Elements of acoustic phonetics. University of Chicago Press; 1996.

[48] Garvin PL, Ladefoged P. Speaker identification and message identification in speech recognition. Phonetica. 1963;9(4):193–199. 10.1159/000258404

[49] Recasens D, Espinosa A. Dispersion and variability of Catalan vowels. Speech communication. 2006;48(6):645–666. 10.1016/j.specom.2005.09.011

[50] Sussman HM. Acoustic correlates of the front/back vowel distinction: a comparison of transition onset versus ‘‘steady state’’. The Journal of the Acoustical Society of America. 1990;88(1):87–96. 10.1121/1.399848 2380450

[51] Schwartz JL, Boë LJ, Vallée N, Abry C. Major trends in vowel system inventories. Journal of Phonetics. 1997;25(3):233–253. 10.1006/jpho.1997.0044

[52] Schwartz JL, Boë LJ, Vallée N, Abry C. The dispersion-focalization theory of vowel systems. Journal of phonetics. 1997;25(3):255–286. 10.1006/jpho.1997.0043

[53] Lindblom B. Explaining phonetic variation: A sketch of the H&H theory In: Speech production and speech modelling. Springer; 1990 p. 403–439.

[54] Lindblom B. Models of phonetic variation and selection. Phonetic Experimental Research, Institute of Linguistics (PERILUS XI), Institute of Linguistics, University of Stockholm. 1990;11:65–100.

[55] Lindblom B. Explaining Phonetic Variation: A Sketch of the H&H Theory In: Speech Production and Speech Modelling. Springer Netherlands; 1990 p. 403–439.

[56] Burzio L. Phonology and phonetics of English stress and vowel reduction. Language Sciences. 2007;29(2-3):154–176. 10.1016/j.langsci.2006.12.019

[57] Barbosa PA, Eriksson A, Åkesson J. Cross-linguistic similarities and differences of lexical stress realisation in Swedish and Brazilian Portuguese. In: Nordic Prosody. Proceedings of the XIth conference. Frankfurt am Main: Peter Lang, Tartu; 2013. p. 97–106. Available from: https://www.isca-speech.org/archive/archive_papers/interspeech_2013/i13_0282.pdf.

[58] Dilley LC, Brown M. Effects of pitch range variation on f0 extrema in an imitation task. Journal of Phonetics. 2007;35(4):523–551. 10.1016/j.wocn.2007.01.003

[59] Gordon M, Roettger T. Acoustic correlates of word stress: A cross-linguistic survey. Linguistics Vanguard. 2017;3(1). 10.1515/lingvan-2017-0007

[60] Künzel HJ. Beware of the ‘telephone effect’: the influence of telephone transmission on the measurement of formant frequencies. Forensic Linguistics. 2001;8(1):80–99. 10.1558/ijsll.v8i1.80

[61] Passetti RR. O efeito do telefone celular no sinal da fala: uma análise fonético-acústica com implicações para a verificação de locutor em português brasileiro; 2015. Available from: http://repositorio.unicamp.br/bitstream/REPOSIP/271133/1/Passetti_RenataRegina_M.pdf.

